# A Two-Tube Multiplex Reverse Transcription PCR Assay for Simultaneous Detection of Sixteen Human Respiratory Virus Types/Subtypes

**DOI:** 10.1155/2013/327620

**Published:** 2013-08-05

**Authors:** Jin Li, Shunxiang Qi, Chen Zhang, Xiumei Hu, Hongwei Shen, Mengjie Yang, Ji Wang, Miao Wang, Wenbo Xu, Xuejun Ma

**Affiliations:** ^1^Key Laboratory for Medical Virology, Ministry of Health, National Institute for Viral Disease Control and Prevention, Chinese Center for Disease Control and Prevention, No. 155, Changbai Road, Changping District, Beijing 102206, China; ^2^National Institute for Nutrition and Food Safety, Chinese Center for Disease Control and Prevention, Beijing 100050, China; ^3^Hebei Center for Disease Control and Prevention, No. 241, Qingyuan Street, Yuhua District, Shijiazhuang 050000, China

## Abstract

There is a need for the development of a rapid and sensitive diagnosis of respiratory viral pathogens. With an intended application in provincial Centers for Diseases Control and Prevention, in this study, we present a two-tube multiplex RT-PCR assay (two-tube assay) using automatic electrophoresis to simultaneously detect sixteen common respiratory viruses. The specificity and the sensitivity of the assay were tested. The assay could detect 20–200 copies per reaction when each viral type was assayed individually, 2000 copies with 9 premixed viral targets in the multiplexed assay in tube 1, and 200 copies with 8 premixed templates in tube 2. A total of 247 specimens were used to evaluate the two-tube assay, and the results were compared with those obtained from the Luminex xTAG RVP Fast assay. The discordant results were confirmed by sequencing or by the Seeplex RV15 ACE detection kit. There were no false positives, but six false negatives occurred with the two-tube assay. In conclusion, the two-tube assay is demonstrated to have great potential for routine surveillance of respiratory virus infection in China.

## 1. Introduction

Viral respiratory tract infections are a common cause of hospitalization and mortality in infants and young children. Influenza A, influenza B, parainfluenza, human rhinovirus, adenovirus, respiratory syncytial virus, metapneumovirus, and coronavirus are recognized as viral agents associated with respiratory tract infections [1–3]. Therefore, there is a need for a rapid and sensitive differential diagnosis of respiratory viral pathogens to prevent nosocomial infections and to minimize unnecessary antibiotic usage [4–6]. 

Existing standard nonmolecular diagnostic methods such as viral culture and immunofluorescence (DFA) are time-consuming, labor intensive, or have low sensitivity [7, 8]. Accordingly, molecular techniques are increasingly accepted for diagnosis of viral respiratory tract infections. In the last few years, multiplex RT-PCR assays have been developed to detect respiratory viruses [9], and some have been commercialized, such as xTAG RVP from Luminex [10, 11], Multicode PLx RVP from EraGen Biosciences [12], and ResPlex II from Qiagen [13]. 

In our previous study, a multiplex PCR based on the GenomeLab Gene Expression Profiler (GeXP) analyzer had been shown to be effective for the detection of pandemic influenza A H1N1 virus [14], nine serotypes of enteroviruses associated with hand, foot, and mouth disease [15], and sixteen different respiratory virus types/subtypes in a single tube [16]. However, these methods are not likely to be widely adopted in provincial Centers for Disease Control and Prevention due to the limited availability of GeXP equipment. Therefore, in this study, a two-tube multiplex reverse transcription PCR assay (twotube assay) to detect sixteen respiratory viruses based on the amplicon size differences using automated electrophoresis system is described. One tube is used for the simultaneous detection of nine respiratory viruses, including influenza A virus (FluA), influenza B virus (FluB), seasonal influenza A virus subtypes H1N1 (sH1N1), parainfluenza virus type 1 (PIV1), human rhinovirus (HRV), coronavirus subtypes OC43 (CoV OC43), coronavirus subtypes 229E (CoV 229E), coronavirus subtypes HKU1 (CoV HKU1), and adenovirus (Adv). Another tube is used for the simultaneous detection of seven respiratory viruses including parainfluenza virus type 2 (PIV2), parainfluenza virus type 3 (PIV3), respiratory syncytial virus A (RSVA), respiratory syncytial virus B (RSVB), coronavirus subtypes NL63 (CoV NL63), human metapneumovirus (HMPV), and human bocavirus (HBoV). As the two-tube assay uses the QIAxcel automated electrophoresis system, which is accessible in most of provincial Centers for Disease Control and Prevention in China, this two-tube assay may have greater potential for the routine surveillance of respiratory virus infection in China and to improve the capacity for emergency management.

## 2. Materials and Methods

### 2.1. Specimens

A total of 247 nasopharyngeal aspirates were collected in the Children's Hospital of Beijing, China, during June 2008 and March 2010 from hospitalized children under two years of age presenting acute lower respiratory infection (ALRI) syndromes based on clinical measurements recommended by the World Health Organization (WHO). The patients were included in this study according to the following criteria: parenchymal infiltration on chest radiography, dry or moist rale, body temperature above 37.5°C, a normal or low leukocyte count, or lower chest wall indrawing. The mean age of study participants was 13.08 months. Of 247 cases, 149 (60.32%) participants were male and 98 (39.68%) female. A total of 160 (64.78%) participants were from urban areas and 87 (35.22%) were from suburban or rural areas of China (Hebei, Henan, Shanxi, Shandong, and Inner Mongolia). The specimens were collected within 5 to 7 days after symptoms onset. Patients with bacterial infections were excluded. All aspects of the study were performed in accordance with national ethics regulations and approved by the Institutional Review Boards of the Center for Disease Control and Prevention of China. Children's parents were apprised of the study's purpose and of their right to keep information confidential. Written consent was obtained from children's parents.

Total RNA/DNA was extracted from 200 *μ*L of clinical sample using the QIAamp Viral RNA Mini Kit (Qiagen, Hilden, Germany). The extracts were eluted into 50 *μ*L of DNase- and RNase-free water and stored at −80°C.

### 2.2. Primers

Nine pairs of chimeric primers [17] were added to tube 1 to detect nine respiratory viruses, and eight pairs of chimeric primers were added to tube 2 to detect seven respiratory viruses. One pair of internal control primer and one pair of universal primers were added to both tubes. The sequence from human genome RNase P gene was used as an internal control primer for the specimens [16]. The primers sequences, the target genes, the amplicon sizes, and primer working concentrations are listed in Table 1.

### 2.3. Two-Tube Assay and Detection

One-Step RT-PCR Kit (Qiagen, Hilden, Germany) was used for the amplification. A total of 25 *μ*L PCR mixture containing 2 *μ*L of extracted RNA and varied primer concentrations (Table 1) was subjected to the following conditions: 50°C for 30 min, 95°C for 15 min, followed by 10 cycles of 95°C for 30 s, 55°C for 30 s, and 72°C 30 s; 10 cycles of 95°C for 30 s, 65°C for 30 s, 72°C for 30 s; 25 cycles of 95°C for 30 s, 48°C for 30 s, and 72°C for 30 s, and a final incubation of 72°C for 3 min [15], and the reactions were analyzed on the QIAxcel automatic electrophoresis using QIAxcel DNA High-Resolution kit.

### 2.4. Specificity and Sensitivity of the Two-Tube Assay

The specificity of the two-tube assay on all viral targets was tested individually in the two multiplex reactions under the experimental condition as described previously. Cell-cultured virus stocks contained in NATtrol Respiratory Validation Panels 2 (NATRVP-2) (ZeptoMetrix, Buffalo, New York, USA) were used as the positive controls of FluA, FluB, sH1N1, PIV1-3, HRV, Adv, RSVA, RSVB, HMPV, CoV 229E, and CoV OC43 to validate the specificity. Specimens genotyped previously [18, 19] were used as positive controls of HBoV, HKU1, and CoV NL63. 

To determine the analytical sensitivity of the assay, serial dilutions of viral RNA transcripts of all RNA viruses and serial dilutions of the plasmids containing the Adv and HBoV target sequence were analyzed by the two-tube assay. The PCR products were cloned into a pGEM-T vector, which was used to transform DH10B cells. The plasmid DNA was extracted with an E.Z.N.A. Plasmid Mini Kit I (Omega, GA, USA). The RNA copy number was calculated after measuring the concentration of the RNA transcribed *in vitro* using a T7 Large-Scale RNA Production System (Promega, Wisconsin, USA). For the DNA viruses Adv and HBoV, the *in vitro* transcription was omitted. The sensitivity of the two-tube assay was examined by using premixed quantitative viral RNA transcripts or plasmids. The personnel responsible for reading these and other assays were experienced staff with basic training in microbiology and molecular biology. 

### 2.5. Luminex xTAG RVP Fast Assay and Detection

The Luminex xTAG RVP Fast kit (Abbott, Illinois, USA) enables users to detect simultaneously FluA, FluB, RSV, PIV1-4, Adv, HMPV, CoV 229E, NL63, OC43, HKU1, enterovirus (HEV)/HRV, and HBoV. The RNA/DNA extracted from 247 clinical specimens was tested using the Luminex xTAG RVP Fast assay in a 96-well plate format according to the manufacturer's instructions [3, 20]. The plate was analyzed using the Bio-Plex 200 system (Bio-Rad, CA, USA), and the median fluorescence intensity (MFI) was determined. 

### 2.6. Resolution and Confirmation of Discordant Results

The additional targets detected only by the two-tube assay were confirmed by independent PCR and sequencing. The sequencing was performed using T7 and SP6 sequence primers on AB SOLiDTM 4.0 System (Applied Biosystems, USA) and compared with the sequences in GenBank for pathogen identification by using the BLAST algorithm. The additional targets detected only by the Luminex xTAG RVP Fast assay were resolved using remaining extracts with the Seeplex RV15 ACE detection kit (Seegene, Seoul, Korea) [21], which contained A, B, and C sets of primers for detection of 15 respiratory viruses, according to the manufacturer's instructions.

### 2.7. Statistical Analysis

All statistical analyses were performed using Statistical Package for Social Sciences (SPSS) software (version 13.0) for Windows. The *χ* ^2^-test and Fisher's exact test were conducted to measure the detection agreement of two-tube assay with the Luminex xTAG RVP Fast assay and confirmed results.

## 3. Results

### 3.1. Specificity and Sensitivity of the Two-Tube Assay

**Figure 1:**
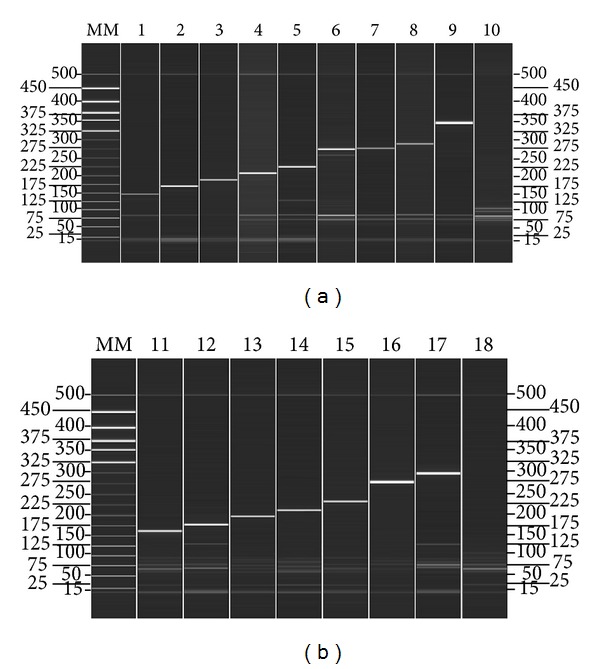
Specificity analyses of the the two-tube assay based on the automatic electrophoresis. All positive controls were identified successfully, and no mispriming was observed in either tube. Lanes 1 to 9 show the results of amplification of HRV (148 bp), FluB (171 bp), CoV 229E (189 bp), CoV OC43 (208 bp), CoV HKU1 (226 bp), FluA (sH1N1) (257 bp and 276 bp), FluA (H3N2) (276 bp), PIV1 (286 bp) and Adv (342 bp), respectively. Lanes 11–17 show the results of amplification of RSVA (163 bp), CoV NL63 (179 b), PIV2 (198 bp), HMPV (212 bp), PIV3 (233 bp), RSVB (280 bp), and HBoV (298 bp), respectively. For the viral targets that used clinical specimens as positive controls, including HBoV, CoV HKU1, and CoV NL63, a single PCR product was detected in addition to the internal control peak (126 bp). Lanes 10 and 18 are the negative controls (distilled water) of tube 1 and tube 2, respectively. Lane MM, molecular marker.

The expected size of each virus type/subtype-specific amplicon was observed and separated clearly from the other viral targets on QIAxcel automatic electrophoresis for all of the positive controls. No mispriming (primer dimer) or other amplification in the negative controls was observed in either tube (Figure 1). The QIAxcel DNA High Resolution kit is capable of resolving amplicons with as little as 5 bp size difference; the smallest size difference in this test was 10 bp. For the viral targets that used clinical specimens as positive controls (HBoV, CoV HKU1, and CoV NL63), a single PCR product was detected in addition to the internal control peak (126 bp). 

The sensitivity of the assay was evaluated for each virus type/subtype individually using serial tenfold dilutions ranging from 10 to 10^5^ copies of cloned PCR products in both of the two tubes. The limit of detection for HRV, PIV2, PIV3, RSVA, HBoV, and Adv was 20 copies per reaction and 200 copies per reaction for the other 10 virus type/subtypes assayed in this study. 

**Figure 2:**
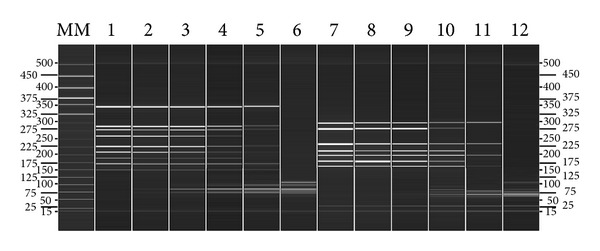
Sensitivity analyses of the two-tube assay based on the automatic electrophoresis. Lanes 1 to 5 contain PCR products of pre-mixed viral targets in tube 1 of 2  ×  10^6^ to 200 copies. Lanes 7 to 11 contain PCR products of pre-mixed viral targets in tube 2 of 2  ×  10^5^ to 20 copies. Lanes 6 and 12 are the negative controls of tube 1 and tube 2, respectively. Lane MM, molecular marker. The detection sensitivity in tube 1 with 9 pre-mixed viral targets was 2000 copies per reaction and 200 copies per reaction in tube 2 with 8 pre-mixed templates. Only the amplicons of sH1N1 and CoV 229E were absent with dilutions of 200 copies of pre-mixed viral targets in tube 1 (Lanes 5), and only the amplicons of CoV NL63, HMPV, and RSVB were absent with dilutions of 20 copies of pre-mixed templates in tube 2 (Lanes 11). The primer dimers which were inevitable in the multiplex PCR are increasingly apparent with the dilution of template.

The sensitivity of two-tube assay when all the viral targets were present was also tested. The detection sensitivity in tube 1 with 9 pre-mixed viral targets was 2000 copies per reaction and 200 copies per reaction in tube 2 with 7 premixed templates (Figure 2).

### 3.2. Evaluation of the Two-Tube Assay Using Respiratory Specimens

All the comparative detections were double-blind tests performed by trained staff in our laboratory. All of the 247 specimens assayed with the Luminex xTAG RVP Fast assay were retested by the two-tube assay. The two assays detected presence of at least one of the 16 assayed viruses in 194 specimens, and the two assays both detected virus in 181 specimens. The two-tube assay detected virus in 8 specimens that were negative in the Luminex xTAG RVP Fast assay (2 Adv, 3 RSVB, 2 HRV, and 1 PIV3), and 5 specimens had a positive detection of HRV only by the Luminex xTAG RVP Fast assay. The Luminex xTAG RVP Fast assay identified 111 specimens to be coinfections, 83 of them (74.77%) were in complete agreement between both assays, and detection of 13 viruses (in 12 specimens) was missed by the two-tube assay. Additional 14 viruses (in 14 coinfected specimens) were detected only by the two-tube assay, and 2 coinfection specimens (no. 28 and no. 118) gave inconsistent results between the two assays (Table 3).

As shown in Table 2, there were 40 viral infections identified only by the two-tube assay and 21 viral infections found only by the Luminex xTAG RVP Fast assay. The infection events not detected by the Luminex xTAG RVP Fast assay included PIV3 (*n* = 2), HRV (*n* = 15), HMPV (*n* = 1), Adv (*n* = 11), RSVA (*n* = 1), and RSVB (*n* = 10). Thirty-two of these undetected targets were found in coinfections, and the remaining 8 targets (2 Adv, 3 RSVB, 2 HRV, and 1 PIV3) were single-target positive specimens by the two-tube assay. The two-tube assay failed to detect the following viral infections (*n* = 21): sH1N1 (*n* = 2), HRV (*n* = 13), Adv (*n* = 1), and HBoV (*n* = 5). Sixteen of the 21 specimens were coinfections, and the remaining 5 targets were positive only for HRV specimens. Because the HRV primers used in the Luminex xTAG RVP Fast assay were able to amplify both HRV and enterovirus, the specimens positive for HRV detected only by the Luminex xTAG RVP Fast assay could be enteroviruses. The sensitivity, specificity, negative prediction value (NPV), positive prediction value (PPV), the accordance rate, and the kappa value of each virus for the two-tube assay, when compared to the Luminex xTAG RVP Fast assay as a reference, are shown in Table 2.

### 3.3. Resolution and Confirmation of Discordant Results

All of the 40 additional targets detected only by the two-tube assay were confirmed by sequencing as true positives. For those specimens with discordant results by the two-tube and RVP assays, the Seeplex RV15 ACE detection kit was used to resolve the discrepancy (Table 3).

 The confirmed results were defined as the results detected by at least two of the three PCR assays [22, 23], or the results of sequencing. The sensitivity, specificity, negative prediction value (NPV), positive prediction value (PPV), the accordance rate, and the kappa value of each virus for the two-tube assay, when compared to the confirmed results as a reference, are shown in Table 4.

## 4. Discussion

In this study, a novel two-tube assay based on the QIAxcel capillary electrophoresis to replace GeXP has been evaluated for the potential application in routine laboratory testing of respiratory viruses in China. Under optimized working condition, the two-tube assay revealed good specificity without nonspecific amplification using size detection system though conventional validation of specificity is usually done by probe detection, hybridization, nested PCR, and sequencing. The two-tube assay achieved a sensitivity of 20–200 copies per reaction when assayed individually for each virus type/subtype, and 2000 copies when 9 viral targets were present in tube 1 and 200 copies when 7 viral templates were present in tube 2. These results suggest the utility of the two-tube assay for the detection of multiple respiratory virus infections for laboratories not equipped to perform liquid chip-based multiplex assays.

The 247 specimens from hospitalized children showed a high prevalence of infection and co-infection with the common respiratory viral pathogens. HRV was found most frequently, followed by RSVB. The major discrepancies between the two-tube assay introduced in this study and the Luminex xTAG RVP Fast assay were found for the detection of HRV, Adv, HBoV, RSVB, and sH1N1 (kappa  =  0.770, 0.773, 0.907, 0.913, and 0.929, resp.) (Table 2). All of the HRV positive samples including 15 detected only by two-tube assay were confirmed by sequencing to be positive for HRV A. The 13 HRV-positive specimens detected only by the Luminex xTAG RVP Fast assay could be enteroviruses because of the Luminex RVP Fast assay detecting enterovirus (the HEV component of the HEV/HRV). After resolved by the Seeplex RV15 ACE detection kit, three of them were truly discordant. For Adv, seven specimens identified as positive only by the two-tube assay were shown via sequencing to be Adv C, and 4 were Adv B1. Only one Adv-positive specimen was missed by two-tube assay. For HBoV, five specimens detected as positive only by the Luminex xTAG RVP Fast assay were also negative by the Seeplex assay. The other 14 positive samples detected only by two-tube assay (10 RSVB, 2 PIV3, 1 RSVA, and 1 HMPV) were confirmed by sequencing as true positives. For sH1N1, all of the FluA positives were sequenced, and 16 of 20 were sH1N1, the two-tube assay failed to detect 2 of the 16 sH1N1 positives. The detection results of coronaviruses (CoV HKU1, CoV NL63, CoV 229E, and CoV OC43), influenza (FluA, FluB), PIV1, and PIV2 were completely consistent between the two assays. In summary, no false positives were found by the two-tube assay, and a total of 6 false negatives occurred: HRV (*n* = 3), sH1N1 (*n* = 2), and Adv (*n* = 1). The two-tube assay was more sensitive than the Luminex xTAG RVP Fast assay for the detection of HRV A, RSVB, Adv B1, and Adv C. The overall detection rate of the two-tube assay for each virus was comparable to that of the Luminex xTAG RVP Fast assay (kappa  >  0.75) demonstrating the high sensitivity and specificity of the two-tube assay in the analysis of clinical samples.

The two-tube assay, as confirmed by the Luminex xTAG RVP Fast assay, revealed that almost 80% of the respiratory specimens tested were positive for at least one viral infection, and viral coinfections were frequent. The overall co-infection rates of the two-tube assay and the Luminex xTAG RVP Fast assay were 48.58% (120/247) and 44.94% (111/247), respectively. Compared with other similar reports [9, 24–26], the extremely higher co-infection rate found by both assays might be due to the difference in the tested population and possible nosocomial infections in the hospital.

 The routine use of extensive testing by methods such as the two-tube assay should make large studies of the clinical relevance of single and multiple respiratory virus infections more feasible. The clinical relevance of the outcome of multiplex PCR tests for respiratory viruses is not yet fully determined. In one study of children with RSV infection, higher fever, longer hospital stays, and more frequent use of antibiotics were associated with multiple infections [27]. For some agents, such as influenza A and B virus, a positive test may provide the basis for antiviral treatment [28]. It has been suggested that a rapid etiologic diagnosis of viral RTI could reduce unnecessary prescription of antibiotics, but this remains to be shown [28].

Two distinct advantages of the two-tube assay are the short assay time and the low cost. The assay requires 5-6 hours to complete 100 samples, including a half hour to prepare PCR mixture, the whole RT-PCR (3 hours), and the detection on the QIAxcel automatic electrophoresis (12 tests/15 minutes), while using other liquid chip-based assays might take an entire workday [29]. The cost for two-tube assay is $10/test, including the RT-PCR kit and the consumables of detection versus at least $120/test [29] using other liquid chip-based assays [29].

In conclusion, the two-tube assay developed in this study using automatic capillary electrophoresis is a rapid, cost-effective method with high sensitivity and specificity for the detection of respiratory virus infection, and it is demonstrated to have great potential for routine surveillance of respiratory virus infection.

## Figures and Tables

**Table 1:** Primer information.

Primer	Sequence 5′-3′	Gene	Size (bp)	Concentrations^a^
FluA F	AGGTGACACTATAGAATATTCTAACCGAGGTCGAAACG	M	270	75 nM/L
FluA R	GTACGACTCACTATAGGGAACAAAGCGTCTACGCTGCAG			
FLuB F	AGGTGACACTATAGAATAAAAAGRAGATTCATCACAGAGC	M	166	50 nM/L
FLuB R	GTACGACTCACTATAGGGATTCTGCTATTTCAAATGCTTCA			
sH1N1 F	AGGTGACACTATAGAATAGGTATGCTTTTGCAMTGARTAGAGG	HA	250	75 nM/L
sH1N1 R	GTACGACTCACTATAGGGAAAGGGATATTCCTTARTCCTGTARCCAT			
PIV1 F	AGGTGACACTATAGAATATCTCATTATTACCYGGACCAA	HA	284	87.5 nM/L
PIV1 R	GTACGACTCACTATAGGGATCCTGTTGTCGTTGATGTCATA			
PIV2 F	AGGTGACACTATAGAATATCTACACTGCATCAGCCAGC	HA	194	50 nM/L
PIV2 R	GTACGACTCACTATAGGGACCCCTAAAAGAGATGAGCCC			
PIV3 F	AGGTGACACTATAGAATATTGTCAATTATGATGGYTCAATCT	HA	230	75 nM/L
PIV3 R	GTACGACTCACTATAGGGAGACACCCAGTTGTGTTGCAG			
HRV F	AGGTGACACTATAGAATACCCCTGAATGYGGCTAACCT	5′ UTR	144	50 nM/L
HRV R	GTACGACTCACTATAGGGACGGACACCCAAAGTAGTYGGT			
HMPV F1	AGGTGACACTATAGAATACATGCCCACTATAAAAGGTCAG	L	208	100 nM/L
HMPV R1	GTACGACTCACTATAGGGACACCCCAGTCTTTCTTGAAA			
HMPV F2	AGGTGACACTATAGAATAGAGCTAAYAGAGTGCTAAGTGATG	N	208	50 nM/L
HMPV R2	GTACGACTCACTATAGGGAACTTTCTGCTTTGCTTCCTGT			
Adv F	AGGTGACACTATAGAATAGCCSCARTGGKCWTACATGCACATC	Hexon	338	100 nM/L
Adv R	GTACGACTCACTATAGGGACAGCACSCCICGRATGTCAAA			
NL63 F	AGGTGACACTATAGAATATCCCAAATGTGATAGAGCTTTGC	Polym-erase	176	50 nM/L
NL63 R	GTACGACTCACTATAGGGACTGTTAAAACTTGTGCCAACTC			
OC43 F	AGGTGACACTATAGAATAATTGCACCAGGAGTCCCA	N	200	50 nM/L
OC43 R	GTACGACTCACTATAGGGATATCGGTGCCGTACTGGTCT			
229E F	AGGTGACACTATAGAATACTCGGAATCCTTCAAGTGACAGA	N	183	50 nM/L
229E R	GTACGACTCACTATAGGGAACGAGAAGGCTTAGGAGTAC			
HKU1 F	AGGTGACACTATAGAATATATAGTRAAACCTGATATGGCT	N	220	50 nM/L
HKU1 R	GTACGACTCACTATAGGGATACCAAAACACTGTTGAACAT			
RSVA F	AGGTGACACTATAGAATACATCCCCTCTATGCACAACC	F	158	50 nM/L
RSVA R	GTACGACTCACTATAGGGACATGTTTCAGCTTGTGGGAA			
RSVB F	AGGTGACACTATAGAATAAAACGAAGATTTCTGGGCTTC	F	279	100 nM/L
RSVB R	GTACGACTCACTATAGGGATGCGACAGCTCTGTTGATTT			
HBoV F	AGGTGACACTATAGAATAAAGAAAAGGGAGTCCAGAA	NP1	290	100 nM/L
HBoV R	GTACGACTCACTATAGGGACTCTGTGTTGACTGAATACAG			
Rnasep F	AGGTGACACTATAGAATAGAGGCCTGGCTTTTGAACTT	RNase-P	125	25 nM/L
Rnasep R	GTACGACTCACTATAGGGAATCAAATTGAGGGCACTGGA			
Tag F	AGGTGACACTATAGAATA			50 nM/L
Tag R	GTACGACTCACTATAGGGA			

^a^Varied primer concentrations in 25 *μ*L PCR mixture.

**Table 2:** Performance of the two-tube assay for individual target compared with the Luminex xTAG RVP Fast assay.

Viruses	No. of specimens: two-tube/Luminex xTAG RVP Fast assay				Performance of the two-tube assay compared with the Luminex xTAG RVP Fast assay					
	+/+	+/−	−/+	−/−	Sensitivity%	Specificity%	PPV%	NPV%	Accordance rate%	Kappa
FluA	20	0	0	227	100	100	100	100	100	1.000
sH1N1	14	0	2	231	87.50	100	100	99.14	99.19	0.929
FluB	1	0	0	246	100	100	100	100	100	1.000
PIV1	1	0	0	246	100	100	100	100	100	1.000
PIV2	2	0	0	245	100	100	100	100	100	1.000
PIV3	22	2	0	223	100	99.11	91.67	100	99.19	0.952
HRV	95	15	13	124	87.96	89.21	86.36	90.51	88.66	0.770
HMPV	27	1	0	219	100	99.55	96.43	100	99.60	0.980
Adv	24	11	1	211	96.00	95.05	68.57	99.53	95.14	0.773
CoV NL63	1	0	0	246	100	100	100	100	100	1.000
CoV OC43	11	0	0	236	100	100	100	100	100	1.000
CoV 229E	2	0	0	245	100	100	100	100	100	1.000
CoV HKU1	3	0	0	244	100	100	100	100	100	1.000
RSVA	7	1	0	239	100	99.58	87.50	100	99.60	0.931
RSVB	86	10	0	151	100	93.79	89.58	100	95.95	0.913
HBoV	28	0	5	214	84.85	100	100	97.72	97.98	0.907

The numbers of positive and negative specimens detected by both testing methods are shown. This table shows the sensitivity, specificity, positive predictive value (PPV), negative predictive value (NPV), and the kappa values for each target using the Luminex xTAG RVP Fast assay as the reference for comparison. All the kappa values were above 0.75, indicating that the two assays were in agreement.

**Table 3:** The confirmed results for specimens with discordant results between the two-tube assay and the Luminex xTAG RVP Fast assay.

Case no.	Two-tube	Luminex xTAG RVP Fast^a^	Seeplex	Confirmed results
43	HRV	HEV/HRV, **HBoV**	HRV	HRV
349	HRV, RSVB	HEV/HRV, RSVB, and **HBoV**	HRV, RSVB	HRV, RSVB
352	HRV, RSVB	HEV/HRV, RSVB, and **HBoV**	HRV, RSVB	HRV, RSVB
146	HMPV	HMPV,** HBoV**	HMPV	HMPV
149	HMPV	HMPV, **HEV**/**H** **R** **V** ^b^, and **HBoV**	HRV, HMPV	**HRV**, HMPV
16	None	**HEV/HRV**	HRV	**HRV**
125	RSVB	RSVB,** HEV/HRV**	HRV, RSVB	**HRV**, RSVB
262	RSVB, PIV3, and HBoV	RSVB, PIV3, HBoV, and** HEV/HRV**	PIV3, RSVB	RSVB, PIV3, and HBoV
270	PIV3, RSVB, and HMPV	PIV3, RSVB, HMPV, and** HEV/HRV**	PIV3, RSVB, and HMPV	PIV3, RSVB, and HMPV
142	RSVB	RSVB, **HEV/HRV**	RSVB	RSVB
21	PIV3	PIV3, **HEV/HRV**	PIV3	PIV3
66	None	**HEV/HRV**	None	None
85	FluA, sH1N1, and HBoV	FluA (H1), HBoV, and **HEV/HRV**	FluA	FluA, sH1N1, and HBoV
87	None	**HEV/HRV **	None	None
91	None	**HEV/HRV**	None	None
114	None	**HEV/HRV**	None	None
274	HRV, RSVB	HEV/HRV, RSVB, and **Adv**	HRV, Adv	HRV, **Adv**, and RSVB
28	FluA, HBoV, and **A** **d** **v** ^c^	FluA **(** **H**1^c^ **)**, HBoV	ND^d^	FluA, **sH1N1**, HBoV, and Adv
118	FluA, Adv, **R** **S** **V** **A** ^c^, **R** **S** **V** **B** ^c^, and HBoV	FluA **(** **H**1^c^ **)**, **HEV/HRV**, Adv, and HBoV	FluA, RSVA, Adv, and HBOV	FluA,** sH1N1**, Adv, RSVA, RSVB, and HBoV

^a^Viruses with discordant results are shown in boldface.

^b^The Luminex xTAG RVP Fast assay was not able to distinguish rhinovirus from enterovirus, so HRV positives are described as HEV/HRV.

^c^The Adv in no. 28 specimen, the RSVA, RSVB in no. 118 specimen, and the FluA subtype H1 were confirmed by sequencing.

^d^ND stands for not detected.

**Table 4:** Performance of the two-tube assay for individual target compared with the confirmed results.

Viruses	No. of specimens by two-tube/confirmed results				Performance of the two-tube assay compared with confirmed results					
	+/+	+/−	−/+	−/−	Sensitivity %	Specificity %	PPV %	NPV %	Accordance rate%	Kappa
FluA	20	0	0	227	100	100	100	100	100	1.000
sH1N1	14	0	2	231	87.50	100	100	99.14	99.19	0.929
FluB	1	0	0	246	100	100	100	100	100	1.000
PIV1	1	0	0	246	100	100	100	100	100	1.000
PIV2	2	0	0	245	100	100	100	100	100	1.000
PIV3	24	0	0	223	100	100	100	100	100	1.000
HRV	110	0	3	134	97.35	100	100	97.81	98.79	0.975
HMPV	28	0	0	219	100	100	100	100	100	1.000
Adv	35	0	1	211	97.22	100	100	99.53	99.60	0.984
CoV NL63	1	0	0	246	100	100	100	100	100	1.000
CoV OC43	11	0	0	236	100	100	100	100	100	1.000
CoV 229E	2	0	0	245	100	100	100	100	100	1.000
CoV HKU1	3	0	0	244	100	100	100	100	100	1.000
RSVA	8	0	0	239	100	100	100	100	100	1.000
RSVB	96	0	0	151	100	100	100	100	100	1.000
HBoV	28	0	0	219	100	100	100	100	100	1.000

This table shows the sensitivity, specificity, positive predictive value (PPV), negative predictive value (NPV), and the kappa values for each target using the confirmed results as the reference for comparison. All the specificity and PPV were 100%, all the accordance rate values were above 98.79%, and all the kappa values were above 0.75.
